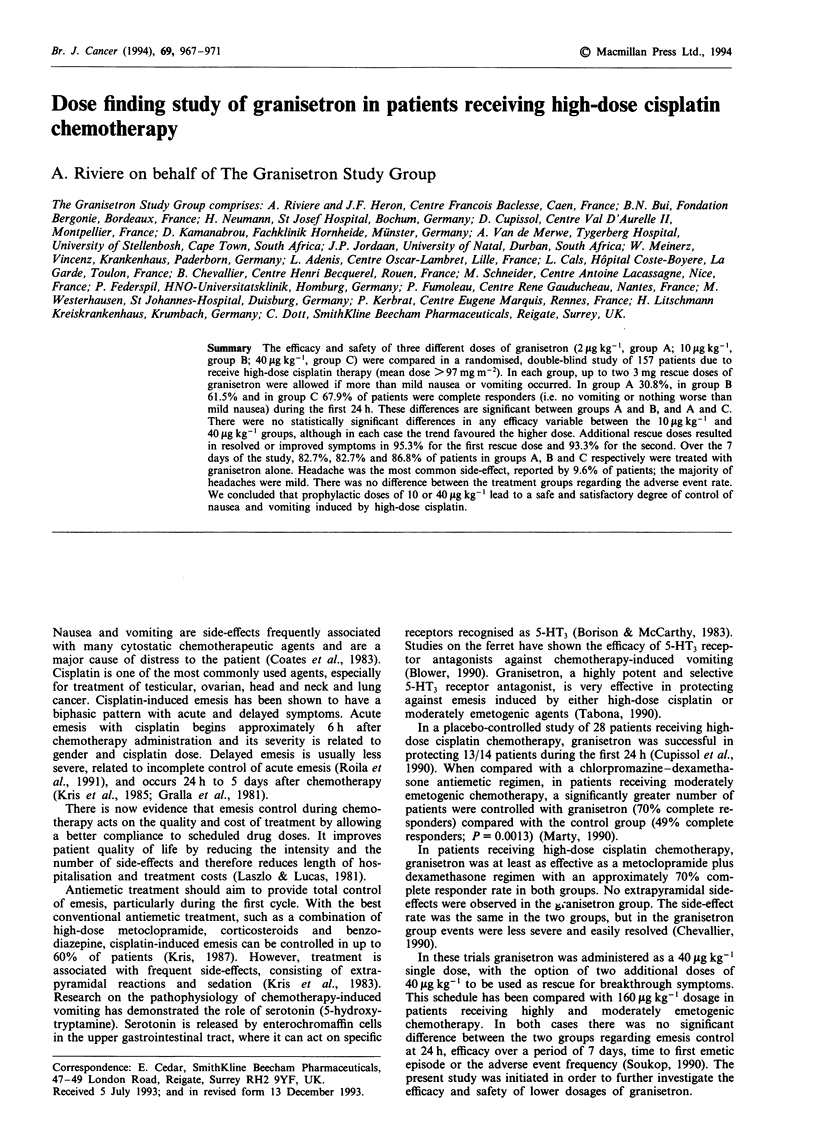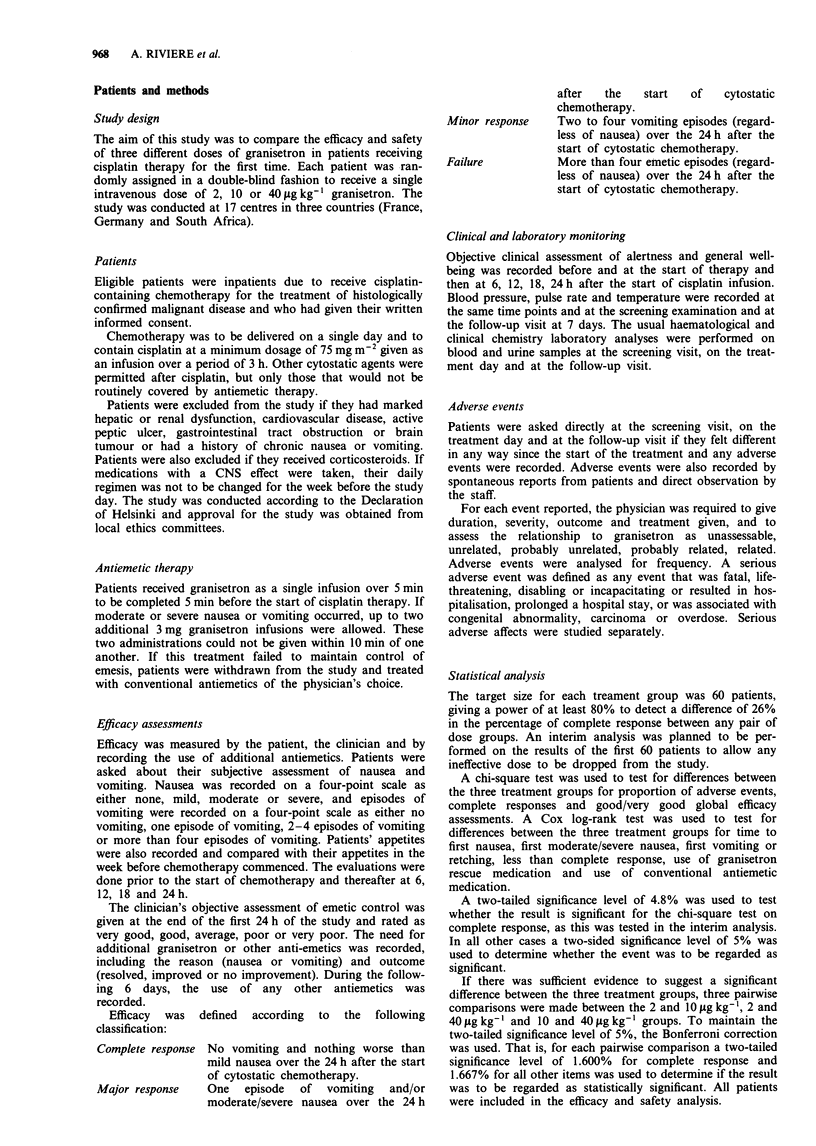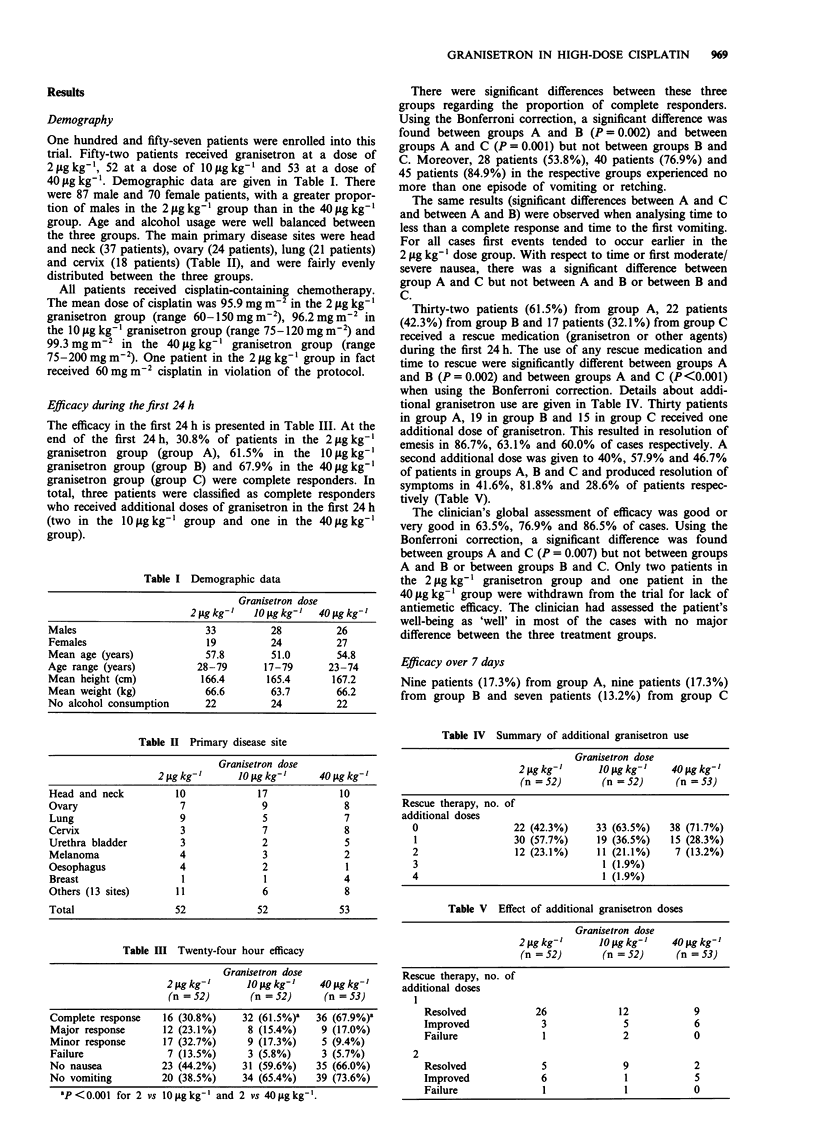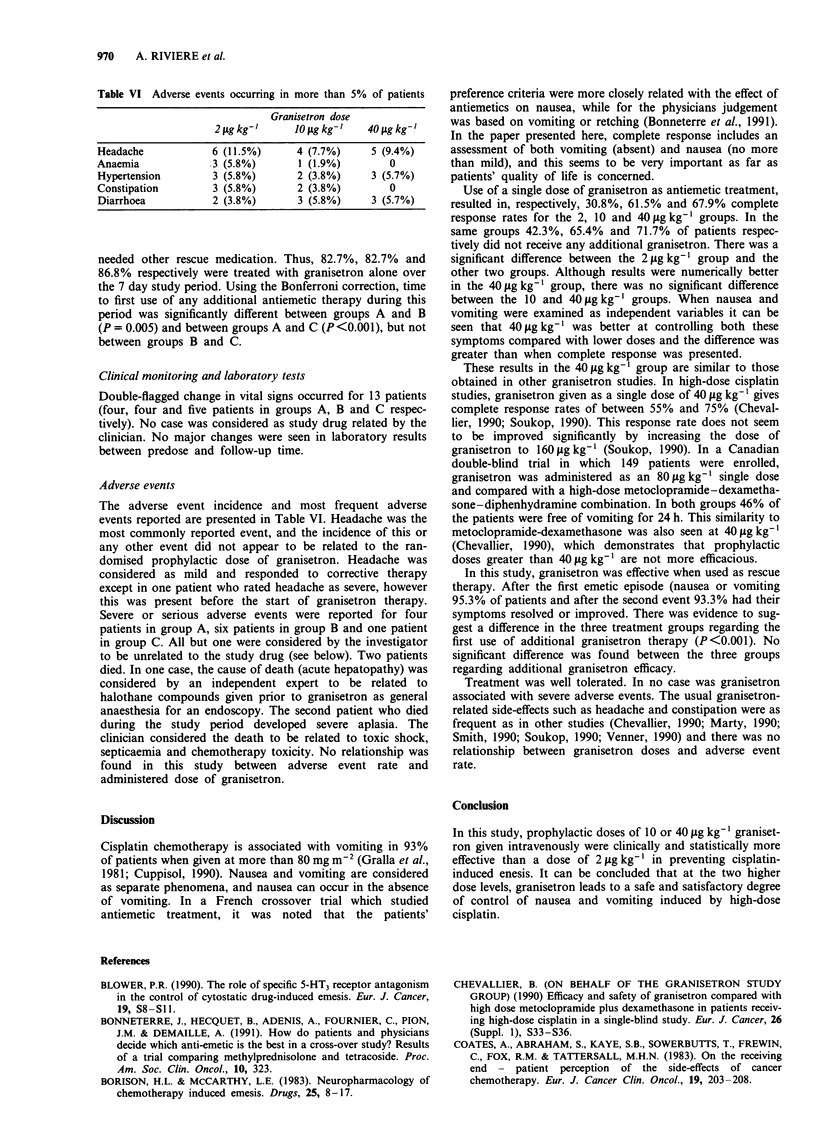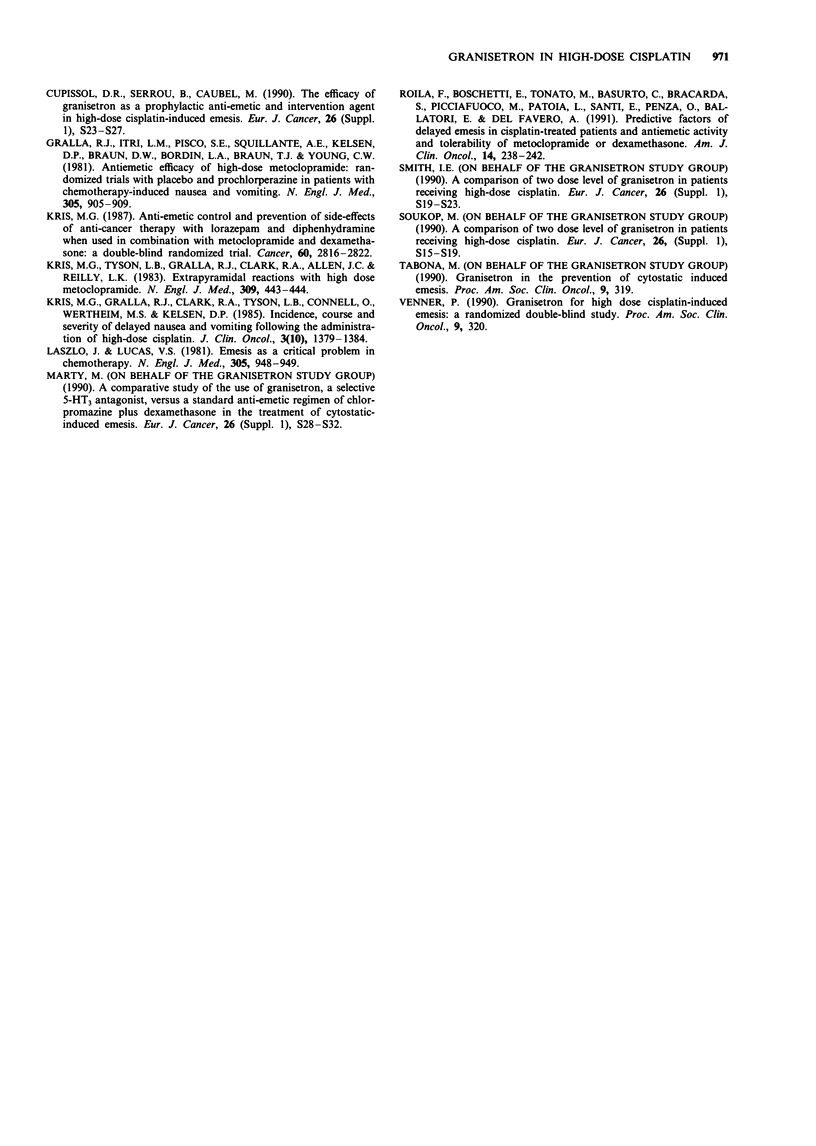# Dose finding study of granisetron in patients receiving high-dose cisplatin chemotherapy. The Granisetron Study Group.

**DOI:** 10.1038/bjc.1994.187

**Published:** 1994-05

**Authors:** A. Riviere

**Affiliations:** Centre Francois Baclesse, Caen, France.

## Abstract

The efficacy and safety of three different doses of granisetron (2 micrograms kg-1, group A; 10 micrograms kg-1, group B; 40 micrograms kg-1, group C) were compared in a randomised, double-blind study of 157 patients due to receive high-dose cisplatin therapy (mean dose > 97 mg m-2). In each group, up to two 3 mg rescue doses of granisetron were allowed if more than mild nausea or vomiting occurred. In group A 30.8%, in group B 61.5% and in group C 67.9% of patients were complete responders (i.e. no vomiting or nothing worse than mild nausea) during the first 24 h. These differences are significant between groups A and B, and A and C. There were no statistically significant differences in any efficacy variable between the 10 micrograms kg-1 and 40 micrograms kg-1 groups, although in each case the trend favoured the higher dose. Additional rescue doses resulted in resolved or improved symptoms in 95.3% for the first rescue dose and 93.3% for the second. Over the 7 days of the study, 82.7%, 82.7% and 86.8% of patients in groups A, B and C respectively were treated with granisetron alone. Headache was the most common side-effect, reported by 9.6% of patients; the majority of headaches were mild. There was no difference between the treatment groups regarding the adverse event rate. We concluded that prophylactic doses of 10 or 40 micrograms kg-1 lead to a safe and satisfactory degree of control of nausea and vomiting induced by high-dose cisplatin.


Br. J. Cancer (1994), 69, 967–971 © Macmillan Press Ltd., 1994

### Dose finding study of granisetron in patients receiving high-dose cisplatin chemotherapy

A. Riviere on behalf of The Granisetron Study Group

*The Granisetron Study Group comprises: A. Riviere and J.F. Heron, Centre Francois Baclesse, Caen, France; B.N. Bui, Fondation Bergonie, Bordeaux, France; H. Neumann, St Josef Hospital, Bochum, Germany; D. Cupissol, Centre Val D'Aurelle II, Montpellier, France; D. Kamanabrou, Fachklinik Hornheide, Münster, Germany; A. Van de Merwe, Tygerberg Hospital, University of Stellenbosh, Cape Town, South Africa; J.P. Jordaan, University of Natal, Durban, South Africa; W. Meinerz, Vincenz, Krankenhaus, Paderborn, Germany; L. Adenis, Centre Oscar-Lambret, Lille, France; L. Cals, Hôpital Coste-Boyere, La Garde, Toulon, France; B. Chevallier, Centre Henri Becquerel, Rouen, France; M. Schneider, Centre Antoine Lacassagne, Nice, France; P. Federspil, HNO-Universitatsklinik, Homburg, Germany; P. Fumoleau, Centre Rene Gauducheau, Nantes, France; M. Westerhausen, St Johannes-Hospital, Duisburg, Germany; P. Kerbrat, Centre Eugene Marquis, Rennes, France; H. Litschmann Kreiskrankenhaus, Krumbach, Germany; C. Dott, SmithKline Beecham Pharmaceuticals, Reigate, Surrey, UK.*

**Summary** The efficacy and safety of three different doses of granisetron ($2\,\mu g\,kg^{-1}$, group A; $10\,\mu g\,kg^{-1}$, group B; $40\,\mu g\,kg^{-1}$, group C) were compared in a randomised, double-blind study of 157 patients due to receive high-dose cisplatin therapy (mean dose $>97\,mg\,m^{-2}$). In each group, up to two 3 mg rescue doses of granisetron were allowed if more than mild nausea or vomiting occurred. In group A 30.8%, in group B 61.5% and in group C 67.9% of patients were complete responders (i.e. no vomiting or nothing worse than mild nausea) during the first 24 h. These differences are significant between groups A and B, and A and C. There were no statistically significant differences in any efficacy variable between the $10\,\mu g\,kg^{-1}$ and $40\,\mu g\,kg^{-1}$ groups, although in each case the trend favoured the higher dose. Additional rescue doses resulted in resolved or improved symptoms in 95.3% for the first rescue dose and 93.3% for the second. Over the 7 days of the study, 82.7%, 82.7% and 86.8% of patients in groups A, B and C respectively were treated with granisetron alone. Headache was the most common side-effect, reported by 9.6% of patients; the majority of headaches were mild. There was no difference between the treatment groups regarding the adverse event rate. We concluded that prophylactic doses of 10 or $40\,\mu g\,kg^{-1}$ lead to a safe and satisfactory degree of control of nausea and vomiting induced by high-dose cisplatin.

Nausea and vomiting are side-effects frequently associated with many cytostatic chemotherapeutic agents and are a major cause of distress to the patient (Coates *et al.*, 1983). Cisplatin is one of the most commonly used agents, especially for treatment of testicular, ovarian, head and neck and lung cancer. Cisplatin-induced emesis has been shown to have a biphasic pattern with acute and delayed symptoms. Acute emesis with cisplatin begins approximately 6 h after chemotherapy administration and its severity is related to gender and cisplatin dose. Delayed emesis is usually less severe, related to incomplete control of acute emesis (Roila *et al.*, 1991), and occurs 24 h to 5 days after chemotherapy (Kris *et al.*, 1985; Gralla *et al.*, 1981).

There is now evidence that emesis control during chemotherapy acts on the quality and cost of treatment by allowing a better compliance to scheduled drug doses. It improves patient quality of life by reducing the intensity and the number of side-effects and therefore reduces length of hospitalisation and treatment costs (Laszlo & Lucas, 1981).

Antiemetic treatment should aim to provide total control of emesis, particularly during the first cycle. With the best conventional antiemetic treatment, such as a combination of high-dose metoclopramide, corticosteroids and benzodiazepine, cisplatin-induced emesis can be controlled in up to 60% of patients (Kris, 1987). However, treatment is associated with frequent side-effects, consisting of extrapyramidal reactions and sedation (Kris *et al.*, 1983). Research on the pathophysiology of chemotherapy-induced vomiting has demonstrated the role of serotonin (5-hydroxytryptamine). Serotonin is released by enterochromaffin cells in the upper gastrointestinal tract, where it can act on specific receptors recognised as 5-$HT_3$ (Borison & McCarthy, 1983). Studies on the ferret have shown the efficacy of 5-$HT_3$ receptor antagonists against chemotherapy-induced vomiting (Blower, 1990). Granisetron, a highly potent and selective 5-$HT_3$ receptor antagonist, is very effective in protecting against emesis induced by either high-dose cisplatin or moderately emetogenic agents (Tabona, 1990).

In a placebo-controlled study of 28 patients receiving high-dose cisplatin chemotherapy, granisetron was successful in protecting 13/14 patients during the first 24 h (Cupissol *et al.*, 1990). When compared with a chlorpromazine–dexamethasone antiemetic regimen, in patients receiving moderately emetogenic chemotherapy, a significantly greater number of patients were controlled with granisetron (70% complete responders) compared with the control group (49% complete responders; $P = 0.0013$) (Marty, 1990).

In patients receiving high-dose cisplatin chemotherapy, granisetron was at least as effective as a metoclopramide plus dexamethasone regimen with an approximately 70% complete responder rate in both groups. No extrapyramidal side-effects were observed in the granisetron group. The side-effect rate was the same in the two groups, but in the granisetron group events were less severe and easily resolved (Chevallier, 1990).

In these trials granisetron was administered as a $40\,\mu g\,kg^{-1}$ single dose, with the option of two additional doses of $40\,\mu g\,kg^{-1}$ to be used as rescue for breakthrough symptoms. This schedule has been compared with $160\,\mu g\,kg^{-1}$ dosage in patients receiving highly and moderately emetogenic chemotherapy. In both cases there was no significant difference between the two groups regarding emesis control at 24 h, efficacy over a period of 7 days, time to first emetic episode or the adverse event frequency (Soukop, 1990). The present study was initiated in order to further investigate the efficacy and safety of lower dosages of granisetron.

Correspondence: E. Cedar, SmithKline Beecham Pharmaceuticals, 47–49 London Road, Reigate, Surrey RH2 9YF, UK.
Received 5 July 1993; and in revised form 13 December 1993.

### Patients and methods

### Study design

The aim of this study was to compare the efficacy and safety of three different doses of granisetron in patients receiving cisplatin therapy for the first time. Each patient was randomly assigned in a double-blind fashion to receive a single intravenous dose of 2, 10 or 40 $\mu g\ kg^{-1}$ granisetron. The study was conducted at 17 centres in three countries (France, Germany and South Africa).

### Patients

Eligible patients were inpatients due to receive cisplatin-containing chemotherapy for the treatment of histologically confirmed malignant disease and who had given their written informed consent.

Chemotherapy was to be delivered on a single day and to contain cisplatin at a minimum dosage of 75 $mg\ m^{-2}$ given as an infusion over a period of 3 h. Other cytostatic agents were permitted after cisplatin, but only those that would not be routinely covered by antiemetic therapy.

Patients were excluded from the study if they had marked hepatic or renal dysfunction, cardiovascular disease, active peptic ulcer, gastrointestinal tract obstruction or brain tumour or had a history of chronic nausea or vomiting. Patients were also excluded if they received corticosteroids. If medications with a CNS effect were taken, their daily regimen was not to be changed for the week before the study day. The study was conducted according to the Declaration of Helsinki and approval for the study was obtained from local ethics committees.

### Antiemetic therapy

Patients received granisetron as a single infusion over 5 min to be completed 5 min before the start of cisplatin therapy. If moderate or severe nausea or vomiting occurred, up to two additional 3 mg granisetron infusions were allowed. These two administrations could not be given within 10 min of one another. If this treatment failed to maintain control of emesis, patients were withdrawn from the study and treated with conventional antiemetics of the physician's choice.

### Efficacy assessments

Efficacy was measured by the patient, the clinician and by recording the use of additional antiemetics. Patients were asked about their subjective assessment of nausea and vomiting. Nausea was recorded on a four-point scale as either none, mild, moderate or severe, and episodes of vomiting were recorded on a four-point scale as either no vomiting, one episode of vomiting, 2–4 episodes of vomiting or more than four episodes of vomiting. Patients' appetites were also recorded and compared with their appetites in the week before chemotherapy commenced. The evaluations were done prior to the start of chemotherapy and thereafter at 6, 12, 18 and 24 h.

The clinician's objective assessment of emetic control was given at the end of the first 24 h of the study and rated as very good, good, average, poor or very poor. The need for additional granisetron or other anti-emetics was recorded, including the reason (nausea or vomiting) and outcome (resolved, improved or no improvement). During the following 6 days, the use of any other antiemetics was recorded.

Efficacy was defined according to the following classification:

| | |
|---|---|
| *Complete response* | No vomiting and nothing worse than mild nausea over the 24 h after the start of cytostatic chemotherapy. |
| *Major response* | One episode of vomiting and/or moderate/severe nausea over the 24 h after the start of cytostatic chemotherapy. |
| *Minor response* | Two to four vomiting episodes (regardless of nausea) over the 24 h after the start of cytostatic chemotherapy. |
| *Failure* | More than four emetic episodes (regardless of nausea) over the 24 h after the start of cytostatic chemotherapy. |

### Clinical and laboratory monitoring

Objective clinical assessment of alertness and general well-being was recorded before and at the start of therapy and then at 6, 12, 18, 24 h after the start of cisplatin infusion. Blood pressure, pulse rate and temperature were recorded at the same time points and at the screening examination and at the follow-up visit at 7 days. The usual haematological and clinical chemistry laboratory analyses were performed on blood and urine samples at the screening visit, on the treatment day and at the follow-up visit.

### Adverse events

Patients were asked directly at the screening visit, on the treatment day and at the follow-up visit if they felt different in any way since the start of the treatment and any adverse events were recorded. Adverse events were also recorded by spontaneous reports from patients and direct observation by the staff.

For each event reported, the physician was required to give duration, severity, outcome and treatment given, and to assess the relationship to granisetron as unassessable, unrelated, probably unrelated, probably related, related. Adverse events were analysed for frequency. A serious adverse event was defined as any event that was fatal, life-threatening, disabling or incapacitating or resulted in hospitalisation, prolonged a hospital stay, or was associated with congenital abnormality, carcinoma or overdose. Serious adverse affects were studied separately.

### Statistical analysis

The target size for each treament group was 60 patients, giving a power of at least 80% to detect a difference of 26% in the percentage of complete response between any pair of dose groups. An interim analysis was planned to be performed on the results of the first 60 patients to allow any ineffective dose to be dropped from the study.

A chi-square test was used to test for differences between the three treatment groups for proportion of adverse events, complete responses and good/very good global efficacy assessments. A Cox log-rank test was used to test for differences between the three treatment groups for time to first nausea, first moderate/severe nausea, first vomiting or retching, less than complete response, use of granisetron rescue medication and use of conventional antiemetic medication.

A two-tailed significance level of 4.8% was used to test whether the result is significant for the chi-square test on complete response, as this was tested in the interim analysis. In all other cases a two-sided significance level of 5% was used to determine whether the event was to be regarded as significant.

If there was sufficient evidence to suggest a significant difference between the three treatment groups, three pairwise comparisons were made between the 2 and 10 $\mu g\ kg^{-1}$, 2 and 40 $\mu g\ kg^{-1}$ and 10 and 40 $\mu g\ kg^{-1}$ groups. To maintain the two-tailed significance level of 5%, the Bonferroni correction was used. That is, for each pairwise comparison a two-tailed significance level of 1.600% for complete response and 1.667% for all other items was used to determine if the result was to be regarded as statistically significant. All patients were included in the efficacy and safety analysis.

### Results

### Demography

One hundred and fifty-seven patients were enrolled into this trial. Fifty-two patients received granisetron at a dose of $2\,\mu g\,kg^{-1}$, 52 at a dose of $10\,\mu g\,kg^{-1}$ and 53 at a dose of $40\,\mu g\,kg^{-1}$. Demographic data are given in Table I. There were 87 male and 70 female patients, with a greater proportion of males in the $2\,\mu g\,kg^{-1}$ group than in the $40\,\mu g\,kg^{-1}$ group. Age and alcohol usage were well balanced between the three groups. The main primary disease sites were head and neck (37 patients), ovary (24 patients), lung (21 patients) and cervix (18 patients) (Table II), and were fairly evenly distributed between the three groups.

All patients received cisplatin-containing chemotherapy. The mean dose of cisplatin was $95.9\,mg\,m^{-2}$ in the $2\,\mu g\,kg^{-1}$ granisetron group (range $60-150\,mg\,m^{-2}$), $96.2\,mg\,m^{-2}$ in the $10\,\mu g\,kg^{-1}$ granisetron group (range $75-120\,mg\,m^{-2}$) and $99.3\,mg\,m^{-2}$ in the $40\,\mu g\,kg^{-1}$ granisetron group (range $75-200\,mg\,m^{-2}$). One patient in the $2\,\mu g\,kg^{-1}$ group in fact received $60\,mg\,m^{-2}$ cisplatin in violation of the protocol.

### Efficacy during the first 24 h

The efficacy in the first 24 h is presented in Table III. At the end of the first 24 h, 30.8% of patients in the $2\,\mu g\,kg^{-1}$ granisetron group (group A), 61.5% in the $10\,\mu g\,kg^{-1}$ granisetron group (group B) and 67.9% in the $40\,\mu g\,kg^{-1}$ granisetron group (group C) were complete responders. In total, three patients were classified as complete responders who received additional doses of granisetron in the first 24 h (two in the $10\,\mu g\,kg^{-1}$ group and one in the $40\,\mu g\,kg^{-1}$ group).

**Table I** Demographic data

| | Granisetron dose | | |
|---|---|---|---|
| | *2 μg kg⁻¹* | *10 μg kg⁻¹* | *40 μg kg⁻¹* |
| Males | 33 | 28 | 26 |
| Females | 19 | 24 | 27 |
| Mean age (years) | 57.8 | 51.0 | 54.8 |
| Age range (years) | 28–79 | 17–79 | 23–74 |
| Mean height (cm) | 166.4 | 165.4 | 167.2 |
| Mean weight (kg) | 66.6 | 63.7 | 66.2 |
| No alcohol consumption | 22 | 24 | 22 |

**Table II** Primary disease site

| | Granisetron dose | | |
|---|---|---|---|
| | *2 μg kg⁻¹* | *10 μg kg⁻¹* | *40 μg kg⁻¹* |
| Head and neck | 10 | 17 | 10 |
| Ovary | 7 | 9 | 8 |
| Lung | 9 | 5 | 7 |
| Cervix | 3 | 7 | 8 |
| Urethra bladder | 3 | 2 | 5 |
| Melanoma | 4 | 3 | 2 |
| Oesophagus | 4 | 2 | 1 |
| Breast | 1 | 1 | 4 |
| Others (13 sites) | 11 | 6 | 8 |
| Total | 52 | 52 | 53 |

**Table III** Twenty-four hour efficacy

| | Granisetron dose | | |
|---|---|---|---|
| | *2 μg kg⁻¹ (n = 52)* | *10 μg kg⁻¹ (n = 52)* | *40 μg kg⁻¹ (n = 53)* |
| Complete response | 16 (30.8%) | 32 (61.5%)[a] | 36 (67.9%)[a] |
| Major response | 12 (23.1%) | 8 (15.4%) | 9 (17.0%) |
| Minor response | 17 (32.7%) | 9 (17.3%) | 5 (9.4%) |
| Failure | 7 (13.5%) | 3 (5.8%) | 3 (5.7%) |
| No nausea | 23 (44.2%) | 31 (59.6%) | 35 (66.0%) |
| No vomiting | 20 (38.5%) | 34 (65.4%) | 39 (73.6%) |

[a]$P<0.001$ for 2 *vs* $10\,\mu g\,kg^{-1}$ and 2 *vs* $40\,\mu g\,kg^{-1}$.

There were significant differences between these three groups regarding the proportion of complete responders. Using the Bonferroni correction, a significant difference was found between groups A and B ($P = 0.002$) and between groups A and C ($P = 0.001$) but not between groups B and C. Moreover, 28 patients (53.8%), 40 patients (76.9%) and 45 patients (84.9%) in the respective groups experienced no more than one episode of vomiting or retching.

The same results (significant differences between A and C and between A and B) were observed when analysing time to less than a complete response and time to the first vomiting. For all cases first events tended to occur earlier in the $2\,\mu g\,kg^{-1}$ dose group. With respect to time or first moderate/severe nausea, there was a significant difference between group A and C but not between A and B or between B and C.

Thirty-two patients (61.5%) from group A, 22 patients (42.3%) from group B and 17 patients (32.1%) from group C received a rescue medication (granisetron or other agents) during the first 24 h. The use of any rescue medication and time to rescue were significantly different between groups A and B ($P = 0.002$) and between groups A and C ($P<0.001$) when using the Bonferroni correction. Details about additional granisetron use are given in Table IV. Thirty patients in group A, 19 in group B and 15 in group C received one additional dose of granisetron. This resulted in resolution of emesis in 86.7%, 63.1% and 60.0% of cases respectively. A second additional dose was given to 40%, 57.9% and 46.7% of patients in groups A, B and C and produced resolution of symptoms in 41.6%, 81.8% and 28.6% of patients respectively (Table V).

The clinician's global assessment of efficacy was good or very good in 63.5%, 76.9% and 86.5% of cases. Using the Bonferroni correction, a significant difference was found between groups A and C ($P = 0.007$) but not between groups A and B or between groups B and C. Only two patients in the $2\,\mu g\,kg^{-1}$ granisetron group and one patient in the $40\,\mu g\,kg^{-1}$ group were withdrawn from the trial for lack of antiemetic efficacy. The clinician had assessed the patient's well-being as 'well' in most of the cases with no major difference between the three treatment groups.

### Efficacy over 7 days

Nine patients (17.3%) from group A, nine patients (17.3%) from group B and seven patients (13.2%) from group C

**Table IV** Summary of additional granisetron use

| | Granisetron dose | | |
|---|---|---|---|
| | *2 μg kg⁻¹ (n = 52)* | *10 μg kg⁻¹ (n = 52)* | *40 μg kg⁻¹ (n = 53)* |
| Rescue therapy, no. of additional doses | | | |
| 0 | 22 (42.3%) | 33 (63.5%) | 38 (71.7%) |
| 1 | 30 (57.7%) | 19 (36.5%) | 15 (28.3%) |
| 2 | 12 (23.1%) | 11 (21.1%) | 7 (13.2%) |
| 3 | | 1 (1.9%) | |
| 4 | | 1 (1.9%) | |

**Table V** Effect of additional granisetron doses

| | Granisetron dose | | |
|---|---|---|---|
| | *2 μg kg⁻¹ (n = 52)* | *10 μg kg⁻¹ (n = 52)* | *40 μg kg⁻¹ (n = 53)* |
| Rescue therapy, no. of additional doses | | | |
| 1 | | | |
| Resolved | 26 | 12 | 9 |
| Improved | 3 | 5 | 6 |
| Failure | 1 | 2 | 0 |
| 2 | | | |
| Resolved | 5 | 9 | 2 |
| Improved | 6 | 1 | 5 |
| Failure | 1 | 1 | 0 |

**Table VI** Adverse events occurring in more than 5% of patients

| | Granisetron dose | | |
|---|---|---|---|
| | *2 μg kg$^{-1}$* | *10 μg kg$^{-1}$* | *40 μg kg$^{-1}$* |
| Headache | 6 (11.5%) | 4 (7.7%) | 5 (9.4%) |
| Anaemia | 3 (5.8%) | 1 (1.9%) | 0 |
| Hypertension | 3 (5.8%) | 2 (3.8%) | 3 (5.7%) |
| Constipation | 3 (5.8%) | 2 (3.8%) | 0 |
| Diarrhoea | 2 (3.8%) | 3 (5.8%) | 3 (5.7%) |

needed other rescue medication. Thus, 82.7%, 82.7% and 86.8% respectively were treated with granisetron alone over the 7 day study period. Using the Bonferroni correction, time to first use of any additional antiemetic therapy during this period was significantly different between groups A and B ($P = 0.005$) and between groups A and C ($P<0.001$), but not between groups B and C.

### *Clinical monitoring and laboratory tests*

Double-flagged change in vital signs occurred for 13 patients (four, four and five patients in groups A, B and C respectively). No case was considered as study drug related by the clinician. No major changes were seen in laboratory results between predose and follow-up time.

### *Adverse events*

The adverse event incidence and most frequent adverse events reported are presented in Table VI. Headache was the most commonly reported event, and the incidence of this or any other event did not appear to be related to the randomised prophylactic dose of granisetron. Headache was considered as mild and responded to corrective therapy except in one patient who rated headache as severe, however this was present before the start of granisetron therapy. Severe or serious adverse events were reported for four patients in group A, six patients in group B and one patient in group C. All but one were considered by the investigator to be unrelated to the study drug (see below). Two patients died. In one case, the cause of death (acute hepatopathy) was considered by an independent expert to be related to halothane compounds given prior to granisetron as general anaesthesia for an endoscopy. The second patient who died during the study period developed severe aplasia. The clinician considered the death to be related to toxic shock, septicaemia and chemotherapy toxicity. No relationship was found in this study between adverse event rate and administered dose of granisetron.

### Discussion

Cisplatin chemotherapy is associated with vomiting in 93% of patients when given at more than 80 mg m$^{-2}$ (Gralla *et al.*, 1981; Cuppisol, 1990). Nausea and vomiting are considered as separate phenomena, and nausea can occur in the absence of vomiting. In a French crossover trial which studied antiemetic treatment, it was noted that the patients' preference criteria were more closely related with the effect of antiemetics on nausea, while for the physicians judgement was based on vomiting or retching (Bonneterre *et al.*, 1991). In the paper presented here, complete response includes an assessment of both vomiting (absent) and nausea (no more than mild), and this seems to be very important as far as patients' quality of life is concerned.

Use of a single dose of granisetron as antiemetic treatment, resulted in, respectively, 30.8%, 61.5% and 67.9% complete response rates for the 2, 10 and 40 μg kg$^{-1}$ groups. In the same groups 42.3%, 65.4% and 71.7% of patients respectively did not receive any additional granisetron. There was a significant difference between the 2 μg kg$^{-1}$ group and the other two groups. Although results were numerically better in the 40 μg kg$^{-1}$ group, there was no significant difference between the 10 and 40 μg kg$^{-1}$ groups. When nausea and vomiting were examined as independent variables it can be seen that 40 μg kg$^{-1}$ was better at controlling both these symptoms compared with lower doses and the difference was greater than when complete response was presented.

These results in the 40 μg kg$^{-1}$ group are similar to those obtained in other granisetron studies. In high-dose cisplatin studies, granisetron given as a single dose of 40 μg kg$^{-1}$ gives complete response rates of between 55% and 75% (Chevallier, 1990; Soukop, 1990). This response rate does not seem to be improved significantly by increasing the dose of granisetron to 160 μg kg$^{-1}$ (Soukop, 1990). In a Canadian double-blind trial in which 149 patients were enrolled, granisetron was administered as an 80 μg kg$^{-1}$ single dose and compared with a high-dose metoclopramide–dexamethasone–diphenhydramine combination. In both groups 46% of the patients were free of vomiting for 24 h. This similarity to metoclopramide-dexamethasone was also seen at 40 μg kg$^{-1}$ (Chevallier, 1990), which demonstrates that prophylactic doses greater than 40 μg kg$^{-1}$ are not more efficacious.

In this study, granisetron was effective when used as rescue therapy. After the first emetic episode (nausea or vomiting 95.3% of patients and after the second event 93.3% had their symptoms resolved or improved. There was evidence to suggest a difference in the three treatment groups regarding the first use of additional granisetron therapy ($P<0.001$). No significant difference was found between the three groups regarding additional granisetron efficacy.

Treatment was well tolerated. In no case was granisetron associated with severe adverse events. The usual granisetron-related side-effects such as headache and constipation were as frequent as in other studies (Chevallier, 1990; Marty, 1990; Smith, 1990; Soukop, 1990; Venner, 1990) and there was no relationship between granisetron doses and adverse event rate.

### Conclusion

In this study, prophylactic doses of 10 or 40 μg kg$^{-1}$ granisetron given intravenously were clinically and statistically more effective than a dose of 2 μg kg$^{-1}$ in preventing cisplatin-induced enesis. It can be concluded that at the two higher dose levels, granisetron leads to a safe and satisfactory degree of control of nausea and vomiting induced by high-dose cisplatin.

### References

BLOWER, P.R. (1990). The role of specific 5-HT$_3$ receptor antagonism in the control of cytostatic drug-induced emesis. *Eur. J. Cancer*, **19**, S8–S11.

BONNETERRE, J., HECQUET, B., ADENIS, A., FOURNIER, C., PION, J.M. & DEMAILLE, A. (1991). How do patients and physicians decide which anti-emetic is the best in a cross-over study? Results of a trial comparing methylprednisolone and tetracoside. *Proc. Am. Soc. Clin. Oncol.*, **10**, 323.

BORISON, H.L. & MCCARTHY, L.E. (1983). Neuropharmacology of chemotherapy induced emesis. *Drugs*, **25**, 8–17.

CHEVALLIER, B. (ON BEHALF OF THE GRANISETRON STUDY GROUP) (1990) Efficacy and safety of granisetron compared with high dose metoclopramide plus dexamethasone in patients receiving high-dose cisplatin in a single-blind study. *Eur. J. Cancer*, **26** (Suppl. 1), S33–S36.

COATES, A., ABRAHAM, S., KAYE, S.B., SOWERBUTTS, T., FREWIN, C., FOX, R.M. & TATTERSALL, M.H.N. (1983). On the receiving end – patient perception of the side-effects of cancer chemotherapy. *Eur. J. Cancer Clin. Oncol.*, **19**, 203–208.

CUPISSOL, D.R., SERROU, B., CAUBEL, M. (1990). The efficacy of granisetron as a prophylactic anti-emetic and intervention agent in high-dose cisplatin-induced emesis. *Eur. J. Cancer*, **26** (Suppl. 1), S23–S27.

GRALLA, R.J., ITRI, L.M., PISCO, S.E., SQUILLANTE, A.E., KELSEN, D.P., BRAUN, D.W., BORDIN, L.A., BRAUN, T.J. & YOUNG, C.W. (1981). Antiemetic efficacy of high-dose metoclopramide: randomized trials with placebo and prochlorperazine in patients with chemotherapy-induced nausea and vomiting. *N. Engl. J. Med.*, **305**, 905–909.

KRIS, M.G. (1987). Anti-emetic control and prevention of side-effects of anti-cancer therapy with lorazepam and diphenhydramine when used in combination with metoclopramide and dexamethasone: a double-blind randomized trial. *Cancer*, **60**, 2816–2822.

KRIS, M.G., TYSON, L.B., GRALLA, R.J., CLARK, R.A., ALLEN, J.C. & REILLY, L.K. (1983). Extrapyramidal reactions with high dose metoclopramide. *N. Engl. J. Med.*, **309**, 443–444.

KRIS, M.G., GRALLA, R.J., CLARK, R.A., TYSON, L.B., CONNELL, O., WERTHEIM, M.S. & KELSEN, D.P. (1985). Incidence, course and severity of delayed nausea and vomiting following the administration of high-dose cisplatin. *J. Clin. Oncol.*, **3(10)**, 1379–1384.

LASZLO, J. & LUCAS, V.S. (1981). Emesis as a critical problem in chemotherapy. *N. Engl. J. Med.*, **305**, 948–949.

MARTY, M. (ON BEHALF OF THE GRANISETRON STUDY GROUP) (1990). A comparative study of the use of granisetron, a selective 5-$HT_3$ antagonist, versus a standard anti-emetic regimen of chlorpromazine plus dexamethasone in the treatment of cytostatic-induced emesis. *Eur. J. Cancer*, **26** (Suppl. 1), S28–S32.

ROILA, F., BOSCHETTI, E., TONATO, M., BASURTO, C., BRACARDA, S., PICCIAFUOCO, M., PATOIA, L., SANTI, E., PENZA, O., BALLATORI, E. & DEL FAVERO, A. (1991). Predictive factors of delayed emesis in cisplatin-treated patients and antiemetic activity and tolerability of metoclopramide or dexamethasone. *Am. J. Clin. Oncol.*, **14**, 238–242.

SMITH, I.E. (ON BEHALF OF THE GRANISETRON STUDY GROUP) (1990). A comparison of two dose level of granisetron in patients receiving high-dose cisplatin. *Eur. J. Cancer*, **26** (Suppl. 1), S19–S23.

SOUKOP, M. (ON BEHALF OF THE GRANISETRON STUDY GROUP) (1990). A comparison of two dose level of granisetron in patients receiving high-dose cisplatin. *Eur. J. Cancer*, **26**, (Suppl. 1), S15–S19.

TABONA, M. (ON BEHALF OF THE GRANISETRON STUDY GROUP) (1990). Granisetron in the prevention of cytostatic induced emesis. *Proc. Am. Soc. Clin. Oncol.*, **9**, 319.

VENNER, P. (1990). Granisetron for high dose cisplatin-induced emesis: a randomized double-blind study. *Proc. Am. Soc. Clin. Oncol.*, **9**, 320.